# Ethical Decision-Making in Chilean University Students: Behavioral and Electroencephalographic Evidence from Professional Ethical Dilemmas

**DOI:** 10.3390/bs16050815

**Published:** 2026-05-19

**Authors:** Jorge Vergara-Morales, Brian Matamala, Bastián Retamal, Carlos Gantiva

**Affiliations:** 1 Laboratorio de Neurociencia Social (NeuroLab), Facultad de Salud y Ciencias Sociales, Escuela de Psicología, Universidad de Las Américas, Concepción 4030000, Chile; brian.matamala@edu.udla.cl (B.M.); bastian.alejandro@edu.udla.cl (B.R.); 2 Departamento de Psicología, Facultad de Ciencias Sociales, Universidad de los Andes, Bogotá 111711, Colombia; c.gantiva@uniandes.edu.co

**Keywords:** ethical decision making, dual-process theory, higher education

## Abstract

Ethical decision-making in professional contexts requires integrating behavioral performance and neural processes, as it involves both deliberative and intuitive mechanisms. However, empirical evidence integrating these levels of analysis in professional ethics remains limited. To address this gap, this study examines the association between behavioral responses and electroencephalographic (EEG) activity during ethical–professional decision-making in a sample of Chilean university students. Thirty-two participants completed a computerized task involving personal and impersonal ethical–professional dilemmas related to psychological practice, making binary decisions while reaction times were recorded. EEG data were acquired using an 8-channel OpenBCI Cyton system. Mean EEG amplitude (0–800 ms post-stimulus) was computed for frontal (Fp1, Fp2, F7, F8) and parietal (Cz, Pz, P3, P4) regions of interest. Behavioral outcomes showed that impersonal dilemmas elicited significantly longer reaction times than personal dilemmas, consistent with greater deliberative demands. Trial-level mixed-effects models revealed a systematic frontal–parietal dissociation, where longer decision durations were associated with increased frontal EEG activity and concurrent parietal suppression. These findings support a systematic behavioral–neural association during ethical–professional decision-making, characterized by a frontal–parietal dissociation that reflects the dynamic competition between deliberative and integrative processes. Prolonged responses to impersonal dilemmas indicate greater deliberative demand, requiring extended integration of abstract professional norms. The observed neural pattern extends dual-process accounts of moral cognition and has implications for the design of ethics education programs that cultivate both deliberative and context-sensitive reasoning skills.

## 1. Introduction

Ethical decision-making is a fundamental competency in higher education, as future professionals must navigate situations in which norms, values, and high-impact social consequences converge. In professional practice, ethical decisions are rarely abstract; they often involve uncertainty, social pressure, and conflicting demands, requiring individuals to integrate multiple sources of information under time constraints (Yoder & Decety, 2018; Sun et al., 2025). Traditional approaches to professional ethics education and research tend to privilege a cognitive and rationalist perspective, emphasizing moral reasoning, normative deliberation, and the explicit application of ethical principles. While these processes are important, this emphasis has frequently relegated affective and intuitive processes to a secondary role, despite growing evidence that such processes play a consequential role in real-world decision-making (Everett & Kahane, 2020; Contreras et al., 2021). Contemporary dual-process models of moral cognition posit that ethical judgments arise from the interaction between relatively fast, intuitive mechanisms and slower, deliberative reasoning systems (Greene et al., 2004; Bago et al., 2021). According to these models, intuitive responses are shaped by automatic integration of affective and contextual cues, whereas deliberative responses require controlled evaluation of norms and outcomes. These mechanisms operate on a continuum rather than as isolated modules, with decision outcomes and speed reflecting the dynamic integration of both processes (Bago & De Neys, 2019; Evans & Stanovich, 2013).

Empirical research supports the relevance of decision time as an index of underlying cognitive dynamics during moral judgment. Reaction time (RT) has been shown to vary systematically with moral conflict, ambiguity, and the need to integrate competing norms, with slower decisions reflecting heightened deliberative effort (Suter & Hertwig, 2011; Cushman, 2013). Decision-making research further indicates that neural activity tracks decision difficulty and latency, reflecting the accumulation of evidence and the engagement of control processes (Turner et al., 2018; Cavanagh & Frank, 2014). Electroencephalography (EEG) provides a valuable means of examining these dynamics in real time, thanks to its high temporal resolution. While EEG studies in moral cognition often focus on event-related potentials (ERPs), recent work emphasizes the value of examining the relationship between EEG activity and behavioral indicators of decision effort, such as RT (Fronda et al., 2024; O’Connell et al., 2012). This process-oriented approach is particularly well-suited to ethical decisions in professional contexts, where decision processes unfold over variable durations and involve integrating cognitive control with contextual information.

Despite these theoretical and methodological developments, applied research on ethical decision-making in professional domains remains scarce. Few studies have examined how behavioral performance and neural activity are coupled during ethical–professional judgment in university students preparing for professional practice. This gap hinders a comprehensive understanding of how future professionals process ethical conflicts and limits the development of educational interventions that integrate both deliberative and intuitive dimensions of ethical competence.

To address this gap, the aim of the study is to examine the association between behavioral responses and electroencephalographic (EEG) activity during ethical–professional decision-making in a sample of Chilean university students. Rather than focusing on categorical neural differences between personal and impersonal dilemmas, the study adopts a process-oriented approach to assess whether decision duration is systematically related to neural engagement during ethical judgment. Consistent with dual-process perspectives, we hypothesize that (H1) impersonal ethical–professional dilemmas will be associated with longer reaction times than personal dilemmas, reflecting increased deliberative demands, and that (H2) behavioral performance during ethical decision-making will be significantly associated with EEG activity, such that longer decision times are accompanied by greater neural engagement.

### 1.1. Dual-Process Theory and Neurophysiological Interpretation

Dual-process theory proposes that moral judgments emerge from the interaction between two partially dissociable, yet dynamically integrated, processing systems. The first system is fast, automatic, and intuitive, operating with minimal conscious deliberation and relying on rapid integration of affective and contextual cues. The second system is slower, controlled, and deliberative, supporting reflective evaluation of norms, consequences, and moral principles. Within this framework, moral dilemmas often involve a tension between deontological tendencies (e.g., rejecting direct harm) and utilitarian considerations (e.g., maximizing overall outcomes), reflecting the balance between intuitive and deliberative processes (Everett & Kahane, 2020; Greene et al., 2004).

Early neuroscientific evidence supporting this model showed that personal dilemmas, characterized by direct, immediate, and personally inflicted harm, were associated with increased engagement of neural systems related to affective and motivational processing. In contrast, impersonal dilemmas, where harm occurs indirectly or without direct physical action, were associated with greater engagement of control-related and evaluative processes. While these findings were initially derived from functional neuroimaging, subsequent electrophysiological studies have extended this distinction by demonstrating systematic differences in scalp-level EEG activity associated with intuitive versus deliberative decision dynamics (Fronda et al., 2024; Greene et al., 2001).

Despite its theoretical influence, dual-process theory has been the subject of substantial criticism. A central criticism concerns the strict separation between System 1 and System 2 processes, which, according to critics, imposes an artificial dichotomy on what is fundamentally a continuous, context-sensitive cognitive system (Melnikoff & Bargh, 2018). Background research suggests that the criteria defining each system, such as automaticity, consciousness, or cognitive effort, are not consistently aligned, making the boundaries between systems theoretically ambiguous and difficult to operationalize empirically (Keren & Schul, 2009). Similarly, it is argued that dual-process models often rely on circular definitions, in which the distinction between fast and slow processes is inferred a posteriori from behavioral outcomes rather than established through independent process-level measurements (Melnikoff & Bargh, 2018).

In the specific field of moral cognition, the dual-process explanation has been challenged from both empirical and conceptual perspectives. In this regard, a two-response paradigm has shown that initial intuitive responses often coincide with utilitarian conclusions, challenging the assumption that utilitarian judgments are exclusively the product of slow, deliberative reasoning. These findings indicate that the relationship between process type and moral content is more flexible than originally postulated by dual-process theory (Cushman, 2013). The continuum model conceptualizes intuitive and deliberative processes as poles on a single dimension of regulatory control, with decision-making outcomes varying with situational constraints, experience, and available cognitive resources (Perugini et al., 2025).

From a neurocomputational perspective, evidence accumulation models, such as the drift-diffusion model, offer a mathematically tractable alternative, positing that moral judgments arise from the continuous integration of decision-relevant information toward a threshold, with reaction time and choice jointly determined by a single evidence-accumulation process (Bago et al., 2021; Bago & De Neys, 2019). These models explain the trade-offs between speed and accuracy, as well as individual differences in decision thresholds, without requiring separate processing systems. Connectionist and predictive coding frameworks further suggest that moral cognition emerges from distributed and reciprocal neural computations rather than the sequential output of independent systems (Evans & Stanovich, 2013). Taken together, these alternative perspectives converge on a process-oriented explanation, in which behavioral markers, such as reaction time, reflect the dynamics of evidence integration rather than the dominance of one system over another.

The present study acknowledges these criticisms and adopts a process-oriented interpretation that is broadly compatible with dual-process perspectives while remaining neutral regarding the strict separability of intuitive and deliberative systems. Rather than using the dual-process framework as a categorical explanatory structure, we employ it as a heuristic guide for interpreting the behavioral and neural signals of decision-making dynamics. Reaction time and frontoparietal EEG activity are treated as continuous, process-level indicators that index the degree of deliberative engagement and integrative neuronal recruitment across trials, without presupposing the existence of two discrete processing systems. This approach aligns with contemporary reformulations of dual-process theory that emphasize flexibility, contextual sensitivity, and the continuous nature of cognitive regulation during ethical judgment (Evans & Stanovich, 2013; Cushman, 2013; Perugini et al., 2025).

Within EEG research, frontal and parietal regions of interest (ROIs) have been widely used as functional markers of these interacting processes. Frontal ROIs, particularly those encompassing frontopolar and lateral frontal electrodes (e.g., Fp1, Fp2, F7, F8), have been consistently linked to cognitive control, conflict monitoring, and response regulation during moral decision-making. Increased frontal EEG activity has been associated with extended deliberation, uncertainty, and the need to integrate abstract norms, especially when decisions require inhibiting automatic responses (Huster et al., 2013; Gui et al., 2016). In contrast, parietal ROIs, typically indexed by midline and lateral parietal electrodes (e.g., Cz, Pz, P3, P4), have been implicated in attentional allocation, contextual integration, and the synthesis of decision-relevant information. Rather than reflecting emotional reactivity alone, parietal EEG activity is increasingly interpreted as supporting efficient integration of affective, contextual, and experiential cues, facilitating rapid judgments when relevant information is readily accessible. Empirical work has shown that parietal activity increases in proportion to decisional relevance and evaluative engagement, particularly in morally meaningful contexts (Fronda et al., 2024; Cui & Nakano, 2024).

Recent reviews and empirical studies emphasize that dual-process theory should not be understood as a strict dichotomy between emotion and reason, but rather as a dynamic system in which contextual demands, prior experience, and emotion regulation strategies continuously modulate the relative contributions of intuitive and deliberative processes. This integrative perspective is especially relevant to EEG research, as its high temporal resolution enables examination of how neural engagement evolves across decision-making and covaries with behavioral indicators such as reaction time (Evans & Stanovich, 2013; Turner et al., 2018). Extending this framework to professional ethics highlights the importance of studying ethical decision-making in ecologically valid contexts. Professional judgments require not only explicit reasoning about rules and principles, but also the rapid application of internalized norms, experiential knowledge, and contextual sensitivity. From this perspective, frontal and parietal ROIs provide complementary windows into the neural dynamics supporting ethical–professional decision-making: frontal engagement reflects extended deliberation and control, whereas parietal engagement reflects efficient integration of professional knowledge and situational cues (Fronda et al., 2024). This neuroethical approach underscores the need for educational models that foster both deliberative competence and intuitive, context-sensitive ethical responsiveness (Hughes & Gallant, 2015).

### 1.2. EEG and Behavioral Dynamics in Ethical Decision-Making

Research on ethical decision-making has increasingly incorporated electrophysiological techniques, such as electroencephalography (EEG), due to their high temporal resolution, which enables neural activity to be examined as decision processes unfold in real time. Rather than focusing exclusively on categorical neural differences across dilemma types, recent approaches emphasize examining how neural engagement covaries with behavioral indices of decision dynamics, particularly reaction time (RT). RT has been widely recognized as a sensitive marker of decisional conflict, cognitive effort, and the integration of competing moral considerations, extending beyond the final choice outcome (Bago & De Neys, 2019; Suter & Hertwig, 2011). From this process-oriented perspective, EEG provides an alternative to analyze how neural activity adapts to decisional demands. Decision-making research indicates that neural signals associated with evidence accumulation and control processes track decision latency and difficulty, with longer response times reflecting extended information integration (Pisauro et al., 2017; London et al., 2019). In moral contexts, increased decision duration has been associated with heightened evaluative processing and regulatory engagement (Bago & De Neys, 2019; Cui & Nakano, 2024). Thus, EEG activity during ethical judgment can be interpreted not only in terms of stimulus category (e.g., personal vs. impersonal dilemmas), but also in relation to the intensity and duration of deliberative processing.

Within scalp-level EEG research, frontal and parietal regions of interest (ROIs) have frequently been used as functional markers of interacting decision mechanisms. Frontal electrodes (e.g., Fp1, Fp2, F7, F8) are commonly associated with cognitive control, conflict monitoring, and the regulation of competing response tendencies during complex moral and value-based decisions (Cavanagh & Frank, 2014; Tremel et al., 2016). Increased frontal EEG activity has been linked to extended deliberation, uncertainty, and the integration of abstract norms, particularly when individuals must inhibit automatic responses (Pierrieau et al., 2022; Clairis & Lopez-Persem, 2023). In contrast, parietal electrodes (e.g., Cz, Pz, P3, P4) have been associated with attentional allocation, evaluative engagement, and the context-sensitive integration of decision-relevant information (Cui & Nakano, 2024; Hunt & Hayden, 2017). Rather than reflecting emotional reactivity alone, parietal EEG activity is increasingly interpreted as supporting efficient synthesis of contextual, experiential, and value-related cues, particularly when judgments are formed rapidly through internalized knowledge structures (Fronda et al., 2024). In morally relevant contexts, parietal activation has been shown to relate to evaluative significance and attentional investment rather than to categorical emotional activation per se (Yoder & Decety, 2014).

Contemporary moral neuroscience no longer conceptualizes dual-process theory as a rigid dichotomy between emotion and reason. Instead, ethical decision-making is viewed as a dynamic interaction between intuitive and deliberative mechanisms, with their relative contributions varying with contextual demands, prior experience, and decisional difficulty (Yoder & Decety, 2014; Jiang et al., 2022; Leng et al., 2021). This integrative perspective is appropriately articulated through EEG methodologies that enable examination of how neural engagement covaries with behavioral markers such as RT (Weindel et al., 2025; Nakatani et al., 2021). Accordingly, in the present study, frontal and parietal ROIs are interpreted as complementary indices of neural engagement during ethical–professional decision-making. Rather than assuming fixed neural signatures for personal and impersonal dilemmas, the analysis examines whether neural activity covaries with decision duration, thereby capturing the degree of deliberative and integrative effort required to reach a judgment in professional contexts.

## 2. Materials and Methods

### 2.1. Design Research

The study employed a within-subjects behavioral–electrophysiological design combining an experimental component, manipulation of dilemma type (personal vs. impersonal), and a correlational component examining the association between trial-level reaction time and scalp EEG activity. The experimental component allowed comparison of behavioral and neural responses across dilemma types, whereas the correlational component assessed whether decision duration systematically predicted neural engagement at the trial level. Causal inference regarding the direction of the RT–EEG relationship is accordingly limited. All participants completed both conditions in a controlled laboratory setting, with electroencephalographic (EEG) activity continuously recorded. Behavioral performance was assessed by binary decisions on each dilemma and corresponding reaction times (RTs), which served as an index of decisional processing demands. EEG activity was analyzed at the scalp level using predefined frontal and parietal ROIs. Frontal ROIs (Fp1, Fp2, F7, F8) were selected based on their established involvement in cognitive control, conflict monitoring, and the evaluation of socially relevant actions, processes supported by medial and lateral prefrontal networks (Cavanagh & Frank, 2014; Levy et al., 2023). Parietal ROIs (Cz, Pz, P3, P4) were selected based on their association with attentional allocation, evidence accumulation signals, and the integration of decision-related evidence, reflecting the accumulation of evaluative information leading to a behavioral response (O’Connell et al., 2012; Steinemann et al., 2018).

### 2.2. Participants

A total of 32 psychology students from a university in south-central Chile participated in the study. Participants’ ages ranged from 19 to 28 years, with a mean age of 23.11 years (SD = 2.01 years). 56% of participants were women and 44% were men, recruited through nonprobability convenience sampling. All participants reported normal or corrected vision, no neurological disorders, and no prior use of psychoactive substances.

### 2.3. Instruments

#### 2.3.1. Experimental Stimuli

Twelve ethical–professional dilemmas (six personal and six impersonal) were used, constructed from information collected through focus groups with practicing professionals. The dilemmas represent conflicts encountered in psychology, particularly in clinical, educational, community, and research contexts. Each dilemma included three phases: (a) instructions, (b) presentation of the dilemma (static visual text), and (c) a position question (Yes/No response to the ethically questionable action), Table 1.

#### 2.3.2. EEG Recording

Electroencephalographic recordings were obtained using an 8-channel Cyton OpenBCI system, configured at a sampling rate of 250 Hz and positioned according to the international 10–20 system. All eight available channels were used, recording from the following scalp sites: Fp1, Fp2, F7, F8 (frontal sites), and Cz, Pz, P3, P4 (centro-parietal sites). Frontal electrodes (Fp1, Fp2, F7, F8) were selected for their established roles in cognitive control, conflict monitoring, and regulation of competing response tendencies during complex moral and value-based decisions (Cavanagh & Frank, 2014; Huster et al., 2013). Parietal electrodes (Cz, Pz, P3, P4) were selected based on their association with attentional allocation, evidence accumulation, and the context-sensitive integration of decision-relevant information (Fronda et al., 2024; Cui & Nakano, 2024). Together, these eight sites provide complementary coverage of the frontoparietal network implicated in deliberative and integrative processes during ethical decision-making. An OpenBCI-compatible electrode cap with saline-moistened Ag/AgCl passive electrodes was used to ensure stable contact and maintain controlled impedance levels throughout the recording session. Electrode-skin impedance was verified before each session using the OpenBCI GUI impedance-monitoring function, ensuring all channels remain below 20 kω. Signals were referenced to the Cyton board’s SRB input (A2) and grounded using the BIAS (driven reference) electrode (A1). During preprocessing, signals were re-referenced offline to the average reference, a common approach in low-density EEG studies that reduces the influence of the hardware reference on scalp-level activity. It is acknowledged that averaging in low-density montages (8 channels) may introduce residual bias, as averaging only a few electrodes may not accurately estimate the true common average. This should be considered when interpreting the spatial distribution of observed scalp-level effects (Luck, 2014). EEG data were recorded using LabRecorder 1.17.1, which simultaneously captured continuous EEG signals and event markers generated by PsychoPy 2026.1.3, ensuring precise synchronization between behavioral responses and neural activity.

### 2.4. Procedure

The study was conducted in a laboratory equipped for the task. Each participant completed the procedure individually, following these steps:

1. Admission, informed consent, and EEG preparation.
2. Task instructions and a brief calibration trial to ensure comprehension and response accuracy.
3. Presentation of 12 ethical–professional dilemmas in randomized order:
  - Each dilemma was presented on screen with unlimited viewing time; participants advanced to the next screen by pressing the space bar once they had finished reading.
  - A minimal inter-stimulus interval (ISI) corresponding to a single screen refresh (~16 ms at 60 Hz) was introduced before the decision prompt, providing temporal separation between stimulus events and their associated EEG markers. This brief ISI was implemented to provide a discrete event marker separating the dilemma reading phase from the decision prompt onset, ensuring that the EEG epoch time-locked to the decision question was not contaminated by stimulus-offset activity from the preceding screen. We acknowledge, however, that this minimal interval does not allow full EEG baseline recovery between phases; future designs should incorporate longer fixed intervals (e.g., 500–1000 ms jittered ISI) to achieve cleaner temporal separation.
  - The decision question was then displayed, and participants responded by pressing “Yes” or “No” on the keyboard. Reaction time was recorded from the onset of the decision question.
4. The total task duration was approximately 12–15 min.
5. At the end of the task, EEG electrodes were removed, and a brief debriefing session was conducted.
6. Stimulus presentation and event control were implemented in PsychoPy Builder, which sent synchronized event markers to the EEG recording system to ensure precise alignment between behavioral responses and neural data.

**Figure 1:**
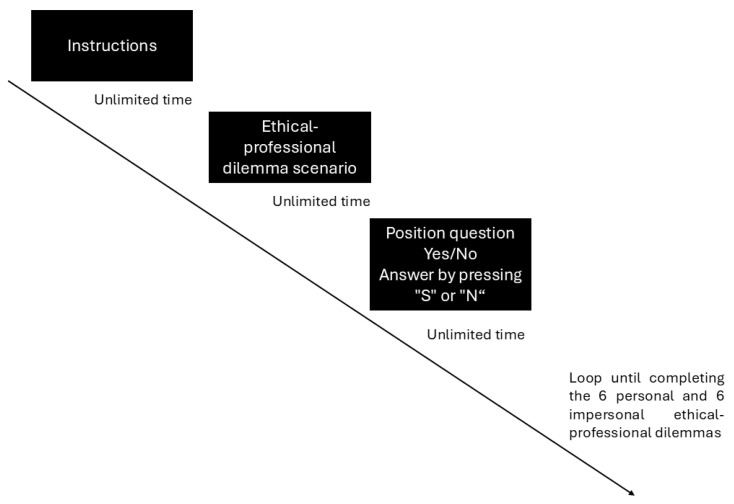
Ethical–professional dilemmas task. Each trial consisted of four sequential phases: (1) general task instructions (unlimited time); (2) presentation of the ethical–professional dilemma scenario as static text (unlimited time; participant advanced by pressing the spacebar); (3) a minimal inter-stimulus interval (~16 ms; single screen refresh at 60 Hz) providing temporal separation between reading and decision phases; and (4) the position question (Yes/No), displayed until response. Reaction time was measured from the onset of the decision question to the key-press response. The loop was repeated for all 12 dilemmas presented in randomized order.

The study adhered to the ethical guidelines of the American Psychological Association (APA), Figure 1.

### 2.5. Data Analyses

#### 2.5.1. EEG Preprocessing

EEG preprocessing was conducted using MNE-Python 1.12.1. Continuous data were band-pass filtered between 0.1 and 30 Hz and re-referenced to the average reference. Data were segmented into epochs time-locked to the onset of the decision question (−200 to 800 ms), and baseline correction was applied using the pre-stimulus interval (−200 to 0 ms). Artifact correction was performed using Independent Component Analysis (ICA; Infomax algorithm). Components corresponding to ocular and muscular artifacts were identified based on scalp topographies, time courses, and spectral properties and were removed prior to signal reconstruction. Epochs exceeding ±100 µV after ICA correction were rejected. After artifact correction and epoch rejection procedures, the distribution of retained epochs was examined across participants and conditions. For personal dilemmas, all epochs were retained across all participants (M = 6.00, SD = 0.00, min = 6, max = 6, n = 32). For impersonal dilemmas, a mean of 5.88 epochs per participant was retained (SD = 0.49, min = 4, max = 6, n = 32), corresponding to an overall retention rate of 97.9% across both conditions. These figures confirm high data quality and consistent signal integrity following preprocessing.

#### 2.5.2. Trial-Level EEG and Behavioral Measures

Behavioral performance was measured by binary responses (Yes/No) and reaction time (RT), recorded for each individual dilemma presentation. EEG activity was analyzed at the single-trial level. For each epoch, the mean EEG amplitude within the post-stimulus interval (0–800 ms) was calculated separately and in combination for the predefined regions of interest (ROIs): frontal (Fp1, Fp2, F7, F8) and parietal (Cz, Pz, P3, P4). The partially overlapping windows were defined to examine the temporal stability of late decision-related neural engagement within the broader 0–800 ms analysis interval rather than to test independent ERP components. This analysis window was selected to capture sustained decision-related neural engagement encompassing late cognitive and evaluative processes, consistent with approaches that index broad post-stimulus neural activity as a correlate of deliberative effort rather than isolating specific ERP subcomponents (Fronda et al., 2024; Weindel et al., 2025). Although this interval does not permit decomposition of temporally distinct neural stages, it provides a robust and theoretically grounded index of overall neural engagement during the decision epoch. Formally, for each trial j of participant i, the mean EEG amplitude within ROI k was computed as A(i,j,k) = (1/T) sum[x(i,j,k,t), t = 1, …, T], where x(i,j,k,t) denotes the baseline-corrected voltage at time sample t for the electrodes assigned to ROI k, and T = 200 samples corresponds to the 800 ms post-stimulus window at a sampling rate of 250 Hz. Channel-level amplitudes were averaged across electrodes within each ROI prior to trial-level analysis. These single-trial neural measures were directly paired with the corresponding trial-level reaction times (RTs), yielding a hierarchical dataset in which each row represented a single dilemma trial nested within participants.

#### 2.5.3. Statistical Analyses

Behavioral differences in RT between personal and impersonal dilemmas were assessed using paired-samples *t*-tests (Langenberg et al., 2023). Effect sizes for paired comparisons were estimated using standardized mean differences based on the average standard deviation of both conditions (Cohen’s d), with Hedges’ correction applied to reduce small-sample bias (g) (Turner et al., 2017). To test neural–behavioral association, linear mixed-effects models were implemented at the trial level, with EEG amplitude as the dependent variable and reaction time as a fixed predictor. Models included random intercepts for participants and dilemmas. The formal model specification for each ROI was: EEGᵢⱼ = β_0_ + β_1_ · log(RT)ᵢⱼ + u_0_ᵢ + εᵢⱼ, where EEGᵢⱼ is the mean amplitude for trial j of participant i, β_0_ is the fixed intercept, β_1_ is the fixed slope for log-transformed reaction time, u_0_ᵢ ~ N(0, σ^2^ᵤ) is the by-participant random intercept, and εᵢⱼ ~ N(0, σ^2^) is the residual error. Models were estimated using restricted maximum likelihood (REML). Separate models were estimated for frontal and parietal ROIs, as well as a combined frontoparietal ROI. Statistical inference was based on the estimated regression coefficients (β), standard errors (SE), z-statistics, and associated *p*-values. To account for multiple testing across ROIs, *p*-values were additionally corrected using both family-wise error correction (pFWE) and false discovery rate correction (pFDR). Effect sizes were quantified using the correlation coefficient (r), and 95% confidence intervals (CI) were reported for parameter estimates. In addition, the marginal coefficient of determination (R^2^m) was computed to estimate the proportion of variance explained by the fixed effects. Because these analyses were defined a priori based on theoretical predictions regarding frontoparietal interactions, they were treated as planned comparisons rather than exploratory multiple tests. This approach allowed assessment of whether longer decision durations were systematically associated with greater neural engagement across trials (Perugini et al., 2025). All preprocessing and statistical analyses were conducted in Python using MNE-Python.

## 3. Results

### 3.1. Behavioral Performance

Reaction times (RTs) were compared between personal and impersonal ethical–professional dilemmas using paired-samples *t*-tests. Results showed a statistically significant difference where impersonal dilemmas elicited longer reaction times than personal dilemmas. Participants responded more slowly to impersonal dilemmas (M = 0.45, SD = 0.21) than to personal dilemmas (M = 0.33, SD = 0.20), reflecting a statistically significant and consistent difference (t_(31)_ = 24.92, *p* < 0.001, Hedges’ g = 0.56, Cohen’s d = 0.57). This effect was consistent across participants, with most individuals showing longer RTs in impersonal dilemmas, indicating pronounced deliberative demands associated with this dilemma type. These behavioral findings are consistent with dual-process accounts of moral decision-making, suggesting that impersonal dilemmas engage slower, more controlled decision-making processes than personal dilemmas (see Table 2).

### 3.2. Behavioral–EEG Association: Trial-Level Mixed-Effects Models

**Figure 2:**
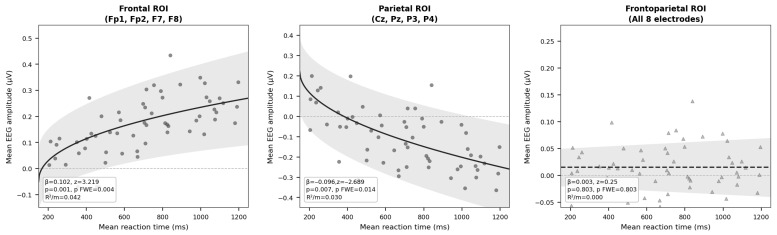
Mean reaction time x mean EEG amplitude. Each data point represents the mean EEG amplitude and mean reaction time for an individual participant × dilemma observation (12 dilemmas per participant). The solid curve indicates the fitted trajectory from the linear mixed-effects model (EEG ~ log(RT) + (1|participant)), with the shaded area representing the 95% confidence interval.

Reaction time emerged as a significant predictor of EEG activity across frontal and parietal regions, revealing a systematic frontal–parietal dissociation. Longer RTs were associated with increased EEG activity in the frontal ROI (Fp1, Fp2, F7, F8; β = 0.102, SE = 0.032, z = 3.22, *p* = 0.001, pFWE = 0.004, r = 0.569, 95% CI [0.040, 0.164], R^2^m = 0.042), indicating greater neural engagement in frontally distributed regions as decision duration increased. In contrast, longer RTs were associated with decreased EEG activity in the parietal ROI (Cz, Pz, P3, P4; β = −0.096, SE = 0.036, z = −2.69, *p* = 0.007, pFWE = 0.014, r = −0.475, 95% CI [−0.165, −0.026], R^2^m = 0.030), suggesting concurrent suppression of parietal activity during more deliberative decisions. Both effects survived family-wise error correction (Holm step-down). The combined frontoparietal ROI yielded no significant effect (β = 0.003, SE = 0.012, z = 0.25, *p* = 0.803), reflecting the opposing directions of the frontal and parietal effects. A combined Fisher test across all three ROI models confirmed global significance, χ^2^_(6)_ = 23.63, *p* < 0.001. Condition (personal vs. impersonal) did not consistently moderate the relationship between RT and EEG activity, indicating that neural engagement was more closely associated with decision duration than with dilemma category per se (see Table 3 and Figure 2). It is important to note that the present analysis examines behavioral–neural covariation through trial-level regression modeling rather than direct functional coupling metrics (e.g., coherence, Granger causality, or dynamic causal modeling). This choice was theoretically motivated: given the low channel density of the recording system and the absence of a priori connectivity hypotheses, spectral coherence or directed influence measures would have limited interpretability. The mixed-effects regression approach provides a well-validated framework for indexing the degree to which neural engagement scales with decision duration at the trial level, and is not intended to imply directional or causal neural coupling (Loewinger et al., 2024).

## 4. Discussion

This study investigated ethical–professional decision-making by integrating behavioral performance and scalp-level electroencephalographic activity in university students. The present study adopted a trial-level EEG approach to investigate decision-related neural engagement during ethical–professional judgment. By linking single-trial neural activity to behavioral indices of decision-making, particularly reaction time, the findings are interpreted as reflecting dynamic evaluative and response-preparatory processes associated with explicit ethical decisions, rather than early moral appraisal during dilemma reading.

Findings indicate that decision duration plays a key role in shaping neural engagement during ethical judgments. Rather than showing strong categorical neural differences between personal and impersonal dilemmas, the results support a process-oriented interpretation in which neural activity adapts to the demands of decision-making (Everett & Kahane, 2020; Bago et al., 2021; Cui & Nakano, 2024). Consistent with hypothesis H1, impersonal dilemmas elicited significantly longer reaction times than personal dilemmas. This pattern suggests that impersonal ethical–professional scenarios require more prolonged deliberative processing, likely reflecting greater engagement with abstract norms, rule-based reasoning, and evaluative integration.

Conversely, personal dilemmas were associated with shorter reaction times, suggesting more streamlined access to internalized professional schemas and context-sensitive evaluative processes that may diminish the need for extended deliberative engagement. Reaction time is widely considered a behavioral marker of decision-related conflict and cognitive demand in moral judgment research (Sun et al., 2025; Cui & Nakano, 2024). Longer decision latencies are typically associated with greater demands on integrative and regulatory processing, rather than simple uncertainty. Thus, the present behavioral findings are consistent with studies proposing that moral decisions vary in their deliberative burden depending on contextual and structural factors.

The main contribution of this study is evidence of a systematic frontal–parietal dissociation linking reaction time to electroencephalographic activity at the trial level, consistent with H2. Longer decision-making processes were associated with increased EEG activity in the frontal ROI (Fp1, Fp2, F7, F8) and, concurrently, with decreased EEG activity in the parietal ROI (Cz, Pz, P3, P4). Both effects survived family-wise error correction and were confirmed by a combined Fisher test across ROI models. The combined frontoparietal ROI showed no significant effect, consistent with opposing frontal and parietal effects rather than reflecting an absence of neural modulation. These findings indicate that neural recruitment varies with decisional effort and is not characterized by category-bound neural signatures. This pattern suggests that electroencephalographic activity during ethical–professional decision-making reflects a dynamic competition between deliberative and integrative systems, rather than a dichotomous activation of distinct processing modules. Neural engagement appears to reorganize as decision duration increases, with frontal regions showing enhanced activity and parietal regions showing concurrent suppression, supporting models of moral cognition that emphasize flexible frontoparietal coordination (Clairis & Lopez-Persem, 2023; Weindel et al., 2025). This finding extends dual-process theory by suggesting that intuitive and deliberative mechanisms do not operate as strictly separable systems, but rather as competing processes whose relative influence shapes the spatiotemporal pattern of neural engagement during ethical judgment.

Frontal scalp activity (Fp1, Fp2, F7, F8) may reflect processes related to cognitive control, evaluative regulation, and conflict monitoring, which become increasingly recruited as decision duration increases (Pierrieau et al., 2022; Clairis & Lopez-Persem, 2023). The positive association between RT and frontal amplitude suggests that more deliberative decisions engage frontal mechanisms more strongly, consistent with evidence linking frontopolar and lateral frontal activity to controlled processing and norm integration (Huster et al., 2013; Gui et al., 2016). In contrast, the negative association between RT and parietal activity (Cz, Pz, P3, P4) suggests that parietal regions undergo suppression during more deliberative decisions. Rather than reflecting simple disengagement, this pattern is consistent with a competitive dynamic in which the increased demands on frontal regulatory systems are accompanied by a relative reduction in parietal activity associated with rapid contextual integration and intuitive appraisal (Fronda et al., 2024; Cui & Nakano, 2024; Jiang et al., 2022; Leng et al., 2021). This interpretation aligns with recent accounts proposing that frontal and parietal contributions to moral judgment are not simply additive but reflect a dynamic rebalancing of deliberative and integrative processes depending on decisional demands (Hunt & Hayden, 2017). Importantly, the null effect in the frontoparietal ROI reflects this opposing pattern: when frontal and parietal signals are averaged together, their opposite polarities cancel, yielding a net-zero effect that masks the underlying dissociation. Thus, the present findings support a model of graded frontoparietal competition rather than uniform frontoparietal coordination during ethical–professional decision-making.

### 4.1. Implications for Ethics Education and Professional Training

The findings carry important implications for ethical and professional education. First, the behavioral differences observed across dilemma types indicate that professional ethical decision-making entails varying deliberative demands. Second, the association between reaction time and neural engagement suggests that ethical competence involves not only knowledge of principles but also the capacity to allocate cognitive resources flexibly in response to situational complexity. The observed frontal–parietal dissociation further suggests that deliberative and integrative processes contribute differentially depending on the demands of the decision, and that ethical training should cultivate both structured reasoning strategies and context-sensitive integrative skills that facilitate rapid appraisal in professional contexts. Ethical training that integrates deliberative reflection with awareness of intuitive processes may better prepare students for real-world professional dilemmas (Contreras et al., 2021).

### 4.2. Limitations and Future Directions

Several limitations should be acknowledged. First, although the sample size is consistent with prior EEG research employing within-subject designs, larger and more diverse samples would enhance statistical power and improve generalizability beyond university students in a single cultural context. Future studies should examine whether similar neural–behavioral association patterns emerge across professional disciplines and cultural settings.

Second, the use of an 8-channel EEG montage limits spatial resolution and precludes source-level inference. Accordingly, interpretations are restricted to scalp-level electrophysiological activity and should not be taken as evidence of specific cortical generators. Because the data were acquired using a low-density montage, spatial interpretations are confined to scalp-level activity patterns, and no source localization or anatomical inference is intended. Future research could incorporate higher-density EEG systems, connectivity analyses, or multimodal neuroimaging approaches to more precisely characterize frontoparietal interactions during ethical–professional decision-making. Additionally, time-frequency decomposition approaches (e.g., wavelet analysis or short-time Fourier transform) could help disentangle early intuitive from late deliberative neural dynamics that are temporally superimposed within the broad 0–800 ms analysis window employed in the present study.

Third, the task included a limited number of dilemmas per condition. Although trial-level mixed-effects modeling increases statistical efficiency by leveraging within-subject variability, future studies would benefit from larger stimulus sets to enhance signal reliability and allow examination of item-level characteristics. Moreover, future research should incorporate psychometrically validated dilemma sets.

Fourth, EEG epochs were time-locked to the onset of the decision question rather than the initial dilemma presentation. This design allowed direct examination of neural activity associated with explicit decision-making, but it does not capture earlier processes during dilemma comprehension. Future paradigms could incorporate controlled reading durations or intermediate markers to analyze comprehension-related and decision-related dynamics.

Fifth, because the design is correlational, the observed associations between reaction time and EEG activity do not permit causal inference regarding whether neural activation drives deliberation or merely reflects it. Future studies could employ experimental manipulations of ethical complexity or cognitive load to more directly evaluate causal mechanisms.

Sixth, the study does not account for individual differences in moral reasoning orientation, professional identity development, or trait-level cognitive styles, factors that may modulate both reaction times and EEG patterns. To explore whether demographic characteristics influenced the observed effects, supplementary analyses were conducted, including sex as a between-participants covariate in the mixed-effects models. These exploratory analyses did not reveal a significant moderation of the RT–EEG relationship by sex (frontal ROI: β_2_ = 0.008, SE = 0.041, z = 0.19, *p* = 0.849; parietal ROI: β_2_ = −0.012, SE = 0.038, z = −0.31, *p* = 0.754), and the primary fixed effects remained substantively unchanged, suggesting that the observed neural-behavioral association was not substantially driven by sex differences in the present sample. Nevertheless, future research incorporating validated measures of moral competence (e.g., Defining Issues Test), empathy (e.g., Interpersonal Reactivity Index), or professional ethical competence would allow more precise interpretation of inter-individual variability.

Seventh, the binary Yes/No response format, while enabling precise RT recording and EEG epoch time-locking, may oversimplify the multidimensional nature of ethical decision-making. Ethical judgment involves not only a final choice but also the integration of value conflicts, professional norms, emotional responses, and contextual reasoning (Everett & Kahane, 2020; Greene et al., 2004). A binary response captures only the direction of the judgment, while omitting potentially meaningful variation in decisional conflict, perceived ethical tension, response certainty, or the weighting of competing professional principles, all of which may systematically influence both reaction time and neural activity. This simplification constrains the interpretability of the observed EEG–RT association, as the full range of deliberative engagement and moral conflict likely exceeds what can be inferred from a forced dichotomy. From a theoretical standpoint, established frameworks such as Rest’s Four Component Model (Narvaez & Rest, 1995) and multidimensional models of professional ethical competence (Johnson et al., 2022) underscore that ethical behavior involves moral sensitivity, judgment, motivation, and implementation, dimensions that cannot be adequately captured by a single binary response. Future research should therefore incorporate methodological approaches that more fully reflect this complexity, including multidimensional rating scales, graded moral conflict measures, confidence ratings, drift-diffusion modeling to decompose decision speed and accuracy, mouse-tracking paradigms to index response competition in real time, or separate assessments of professional values (e.g., autonomy, beneficence, justice). Such approaches would allow examination of whether the frontal–parietal dissociation observed here is modulated by the degree of perceived ethical conflict or decisional certainty, thereby providing a richer characterization of the neural dynamics underlying ethical–professional judgment.

Eighth, because dilemma reading time was self-paced and unlimited, individual differences in comprehension duration may have introduced residual neural activity into the decision epoch. Future designs should incorporate controlled reading windows or model reading duration as a covariate to better isolate decision-specific neural engagement.

Ninth, the study does not evaluate the ethical quality or professional appropriateness of participants’ decisions. Incorporating expert-rated ethical quality scores aligned with professional guidelines (e.g., APA Ethics Code) would allow examination of whether deliberative engagement is associated with more nuanced or professionally appropriate responses. Future research should also extend this paradigm to diverse cultural contexts, professional disciplines (e.g., law, medicine, education), and employ higher-density EEG to better characterize frontoparietal network dynamics and enable source localization.

## 5. Conclusions

The present study provides behavioral and neurophysiological evidence for a systematic frontal–parietal dissociation that characterizes ethical–professional decision-making. Impersonal dilemmas elicited significantly longer reaction times than personal dilemmas, consistent with increased deliberative demands. Scalp-level EEG activity did not reveal fixed categorical neural signatures tied exclusively to dilemma type. Instead, decision duration was associated with increased frontal engagement and concurrent parietal suppression, both of which survived family-wise error correction. This opposing pattern indicates that ethical judgments involve a dynamic competition between deliberative and integrative neural processes, rather than graded co-activation of frontoparietal systems.

These findings extend contemporary dual-process perspectives by emphasizing that ethical decision-making in professional contexts cannot be reduced to a simple opposition between emotion and reason. Rather than reflecting strictly separable systems, the frontal–parietal dissociation suggests that deliberative and integrative mechanisms operate in dynamic competition, with their relative balance shifting as decisional demands increase.

From a practical standpoint, the observed frontal–parietal dissociation underscores the importance of fostering both deliberative reasoning skills and context-sensitive integrative abilities in professional ethics education. Ethical competence involves not only mastery of normative principles but also the capacity to allocate cognitive resources adaptively in response to situational complexity. Training programs that combine case analysis with reflective exercises to increase awareness of the dynamics of the decision-making process can better prepare students to engage effectively in ethically complex professional scenarios. Overall, by integrating behavioral performance and electrophysiological measures within ecologically grounded professional dilemmas, this study contributes to a more nuanced understanding of how neural and behavioral dynamics jointly shape ethical decision-making in future professionals.

## Figures and Tables

**Table 1:** Ethical–professional dilemma case descriptions.

Dilemmas Name	Description
1. Ethics of the Greater Good vs. Professional Truthfulness	An educational psychologist is pressured to alter special educational needs (SEN) diagnostic scores to maximize overall well-being.
2. To Continue or to Refer? The Practitioner’s Conflict of Responsibility	A psychology student in clinical practice believes his approach will not achieve the therapeutic goals and requests a change of patient. However, his supervisor advises that maintaining the therapeutic relationship is key to patient adherence.
3. Confidentiality vs. Protection of Third Parties	A patient expresses intentions to harm a coworker. The psychologist must decide whether to maintain confidentiality or intervene to prevent potential harm to others.
4. To Discharge or to Persist? The Weight of the Relationship in Decision-Making	A patient stops attending therapy, and the protocol dictates discharging them, but the student fears harming a vulnerable patient and feels emotionally responsible for attempting to contact them again.
5. The Dilemma of Therapeutic Continuity in Clinical Training	After four sessions, a student requests a change of patient, indicating that the approach he is proficient in does not allow him to progress. However, his coordinator reminds him that such a change could negatively affect the therapeutic process.
6. To Interfere or to Report: Ethics in the Face of Protocol Activation	A professional close to the family of a student accused of bullying withdraws from the case to avoid a conflict of interest but then meets with the family to inform them of institutional protocol, thereby compromising impartiality.
7. Confidentiality vs. Protection against Imminent Risk	A patient, in a state of intense emotional distress, states that “something serious could happen soon.” Without a clear plan, the psychologist must decide whether to respect confidentiality or act to prevent immediate harm.
8. Deciding When to Stop vs. Continue Attending to a Patient	A student is seeing a vulnerable patient who stops attending sessions. Although the protocol dictates closing the case, the student wants to persist to prevent the patient from being left without psychological support.
9. The Dilemma of Confidentiality in Therapy with Adolescents	A teenager reveals intimate issues and asks the psychologist not to share them with their parents. The professional must decide whether to protect confidentiality or involve the family in the treatment.
10. The Dilemma of Scientific Integrity in the Face of Pressure to Publish	A researcher reflects on excluding “outliers” that render their study’s results insignificant, even at the risk of compromising scientific ethics to advance their academic career.
11. Helping Without Harm: The Dilemma of Professional Competence in an Emergency	After an earthquake, an educational psychologist is asked to provide psychological first aid, but the professional lacks the necessary training. They must decide whether to assist in the crisis or refrain from intervention due to concerns about potential consequences.
12. Ethical Integrity vs. Institutional Pressure in Intervention Reports	A supervisor suggests that students modify narrative fragments or “adjust” examples in reports to demonstrate the program’s impact.

**Table 2:** Behavioral performance across ethical–professional dilemma conditions.

Condition	RT Means	RT SD	% Yes Responses	t (*p*)	Hedges’ g	Cohen’s d
Impersonal	0.45	0.21	59.90	24.92 (*p* < 0.001)	0.56	0.57
Personal	0.33	0.20	22.40			

Note. RT values represent subject-level mean reaction times in seconds.

**Table 3:** Mixed-effects models predicting EEG activity from decision-related variability.

ROI	β	SE	Z	*p*	pFWE	pFDR	r	IC	R^2^m
Frontal	0.102	0.03	3.219	0.001	0.004	0.004	0.57	[0.04, 0.16]	0.042
Parietal	−0.096	0.04	−2.689	0.007	0.014	0.011	−0.48	[−0.17, −0.03]	0.030
Frontoparietal	0.003	0.01	0.250	0.803	0.803	0.803	0.04	[−0.03, 0.03]	0.000

Note. Fixed-effect estimates from trial-level linear mixed-effects models (REML) predicting mean EEG amplitude.

## Data Availability

The data presented in this study are available on request from the corresponding author.
